# 2-{[(1*S*,2*S*)-1-Hy­droxy-1-phenyl­propan-2-yl](methyl)amino}-*N*-(3-methyl­phen­yl)acetamide

**DOI:** 10.1107/S2414314625005784

**Published:** 2025-07-04

**Authors:** Sarvinoz Bobonazarova, Batirbay Torambetov, Dilnoza Burieva, Anvar Abdushukurov, Shakhnoza Kadirova, Rasul Ya Okmanov, Mukhriddin Yusufov

**Affiliations:** a National University of Uzbekistan named after Mirzo Ulugbek 4 University St Tashkent 100174 Uzbekistan; b Physical and Material Chemistry Division CSIR-National Chemical Laboratory,Pune 411008 India; c S.Yunusov Institute of the Chemistry of Plant Substances Academy of Sciences of Uzbekistan Mirzo Ulugbek St 77 Tashkent 100170 Uzbekistan; Howard University, USA

**Keywords:** pseudoephedrine, organic synthesis, chiral centers, 2-chloro­acetamides, crystal structure

## Abstract

The title compound, C_19_H_24_N_2_O_2_ (*m*-TAP), was synthesized *via* the reaction of 2-chloro-*N*-(*m*-tol­yl)acetamide with pseudoephidrine. It crystallizes in the monoclinic crystal system with a non-centrosymmetric chiral space group *P*2_1_ (No. 4). The asymmetric unit comprises a single mol­ecule. The dihedral angle between the phenyl rings is 89.20 (10)°. *m*-TAP contains two chiral centers at the C7 and C8 positions, both with an absolute configuration of *S*.

## Abstract 2

A chiral compound, *m*-TAP, was synthesized and its crystal structure was determined, revealing key stereochemical and supra­molecular features.

## Graphical abstract

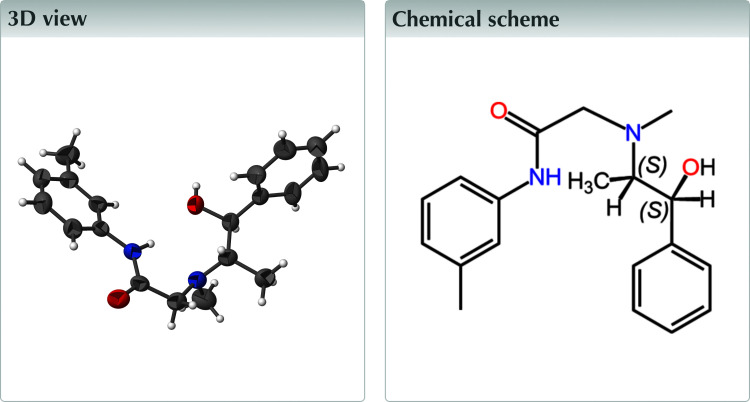

## Structure description

**Figure 1:**
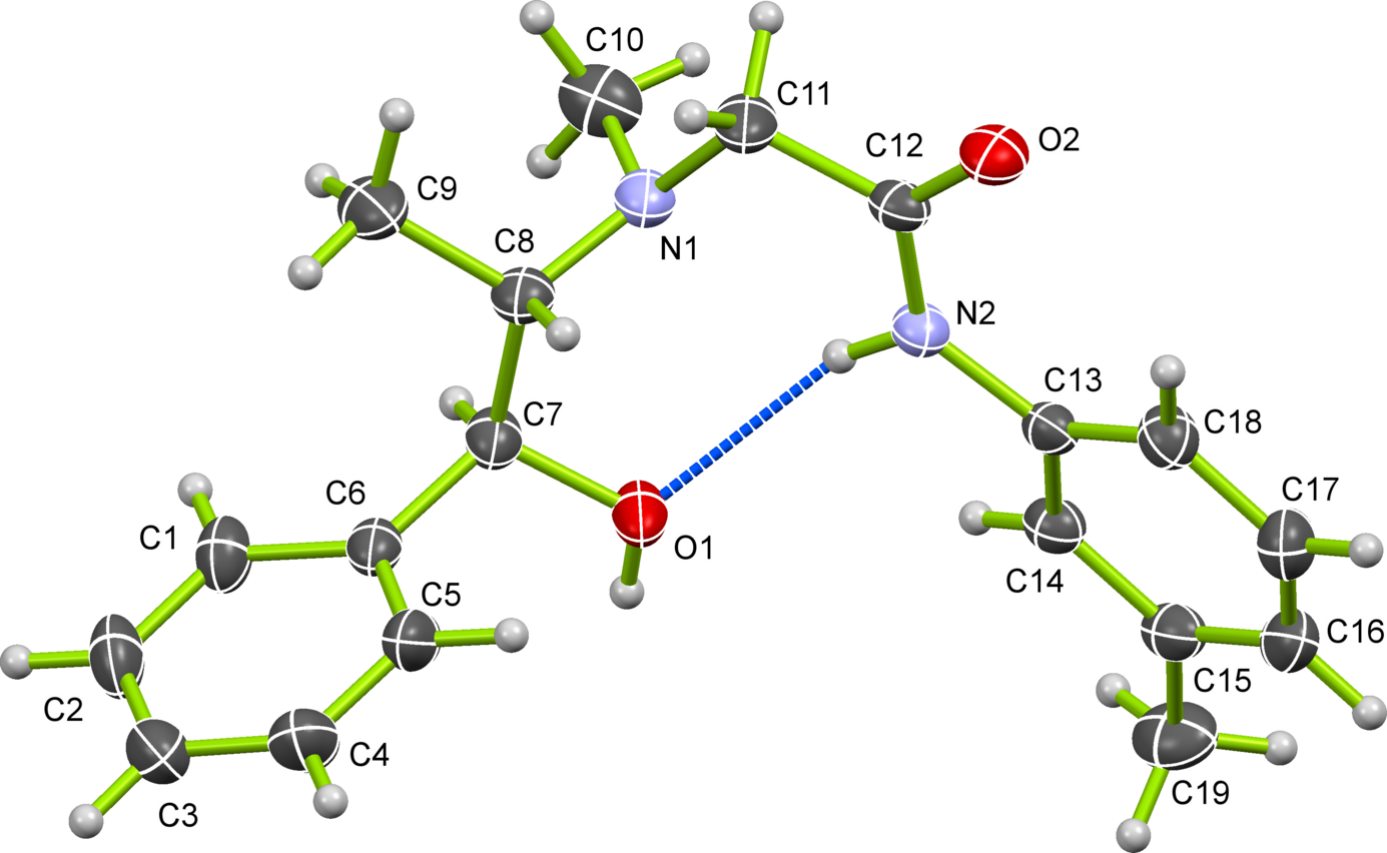
The mol­ecular structure of *m*-TAP showing the atom labeling with displacement ellipsoids drawn at the 30% probability level. Hydrogen atoms are represented as small spheres with arbitrary radii. The intra­mol­ecular hydrogen bond is indicated by a blue dashed line.

**Figure 2:**
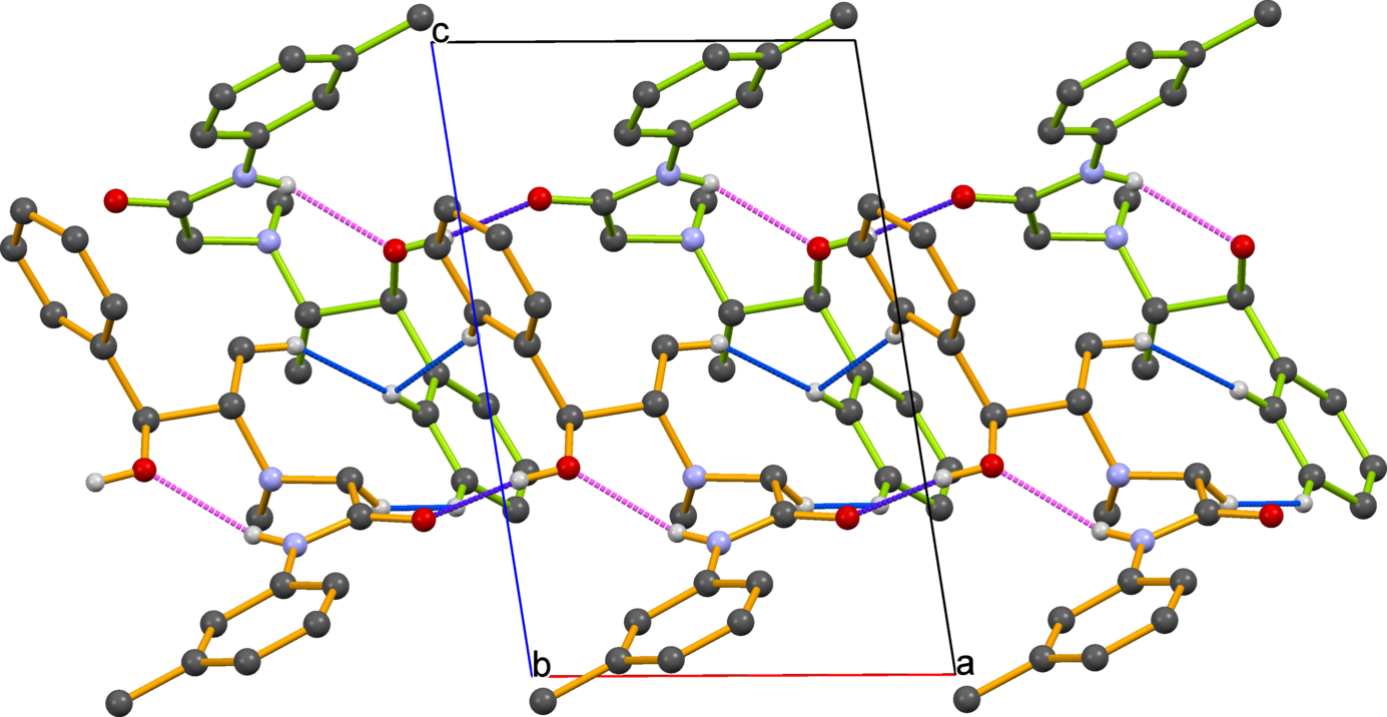
The packing of mol­ecules in *m*-TAP, connected *via* O⋯H and H⋯H inter­actions, viewed along the *b-*axis direction.

Extensive global research is focused on the efficient synthesis of *N*-acetamide-bonded compounds *via* chloro­acetyl­ation of amino compounds, especially aromatic and heterocyclic amines containing heteroatoms (Souza *et al.*, 2019 ▸; Ma *et al.*, 2011 ▸). These aromatic amides are vital inter­mediates in pharmaceuticals (*e.g.*, anesthetics such as mepivacaine, articaine, prilocaine), hypnotics, diuretics (*e.g.*, methaqua­lone), agricultural fungicides (*e.g.*, triazole derivatives), herbicides (*e.g.*, metolachlor), and in the oil and gas industry. In this study, *m*-toluidine was reacted with chloro­acetyl chloride to form 2-chloro-*N*-*m*-tolyl­acetamide, followed by a reactivity study with pseudoephedrine to form *m*-TAP. The crystal structure of *m*-TAP (Fig. 1 ▸) was determined using single-crystal X-ray diffraction (SCXRD) indicating that it crystallizes in the monoclinic crystal system with a non-centrosymmetric chiral space group *P*2_1_ (No. 4). The dihedral angle between the two phenyl rings is 89.20 (1)°, indicating a nearly perpendicular orientation. The torsion angles for the fragments C18—C13—N2—C12 and C5—C6—C7—C8 are 17.0 (3) and −73.9 (2)°, respectively. The twisted mol­ecular conformation prevents effective π–π stacking inter­actions between the benzene rings. Additionally, *m*-TAP possesses two chiral centers at the C7 and C8 positions, with both absolute configurations being S. An intra­molecular hydrogen bond [N2—H2⋯O1, H⋯*A* = 2.23 (3) Å; Table 1 ▸] occurs between the hydrogen atom of the amine group and the carbonyl oxygen atom. In addition, two neighbouring mol­ecules are linked through a strong inter­molecular hydrogen-bonding inter­action [O1—H1⋯O2, H⋯*A* = 1.88 (3) Å; Table 1 ▸] involving the carbonyl oxygen atom and the hydroxyl hydrogen atom of an adjacent mol­ecule. Moreover, the C—H groups of the phenyl ring are involved in H⋯H inter­actions with hydrogen atoms from the methyl group attached to chiral carbon (C8) and meth­ylene (C11) groups, as well as with the phenyl C—H groups of adjacent mol­ecules (Fig. 2 ▸).

## Synthesis and crystallization

**Figure 3:**
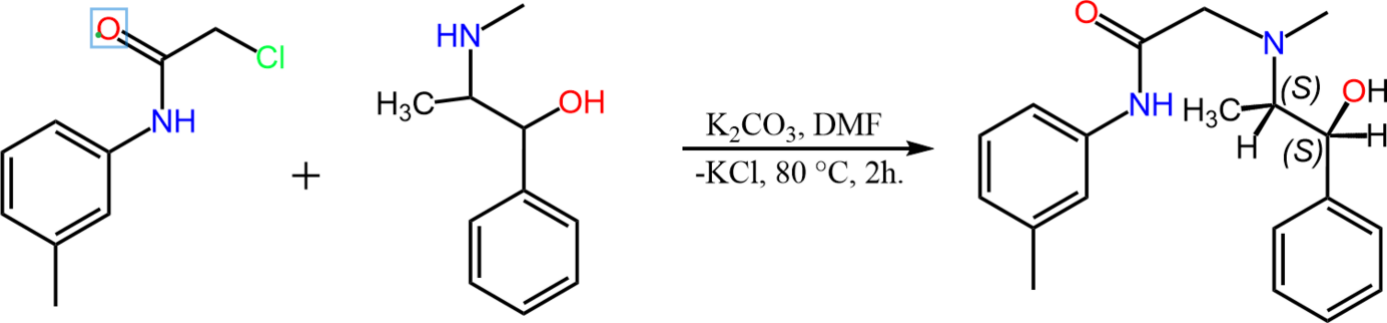
Reaction scheme.

Into a 50 ml round-bottom flask, 0.1835 g (1.0 mmol) of 2-chloro-*N*-*o*-tolyl­acetamide and 0.138 g (1.0 mmol) of potassium carbonate (K_2_CO_3_) were added. To this, 2 ml of *N*,*N*-di­methyl­formamide (DMF) and 0.165 g (1.0 mmol) of pseudoephedrine were added. The reaction mixture was placed in an ultrasonic water bath and stirred at 80°C for 2 h. Reaction progress was monitored every 15 minutes using thin-layer chromatography (TLC), with a mobile phase consisting of hexa­ne:ethyl acetate:methanol in a 1:1:0.25 ratio. After completion, the reaction mixture was poured into 20 ml of ethyl acetate and washed twice with 20 ml of water. The organic layer was dried over anhydrous sodium sulfate (Na_2_SO_4_), and the solvent was removed under reduced pressure using a rotary evaporator. The crude product was recrystallized from acetone to afford a white crystalline solid. Yield: 91%, m.p. 156–157.3°C. The reaction scheme is shown in Fig. 3 ▸.

## Refinement

Crystal data, data collection and structure refinement details are summarized in Table 2 ▸.

## Supplementary Material

Crystal structure: contains datablock(s) I. DOI: 10.1107/S2414314625005784/bv4055sup1.cif

Structure factors: contains datablock(s) I. DOI: 10.1107/S2414314625005784/bv4055Isup2.hkl

Supporting information file. DOI: 10.1107/S2414314625005784/bv4055Isup3.cml

CCDC reference: 2467626

Additional supporting information:  crystallographic information; 3D view; checkCIF report

## Figures and Tables

**Table 1:** Hydrogen-bond geometry (Å, °)

*D*—H⋯*A*	*D*—H	H⋯*A*	*D*⋯*A*	*D*—H⋯*A*
C11—H11*A*⋯C1^i^	0.97	2.83	3.720 (3)	152
C16—H16⋯C12^ii^	0.93	2.80	3.378 (3)	121
C2—H2*A*⋯C14^iii^	0.93	2.86	3.687 (3)	149
C1—H1*A*⋯C5^iii^	0.93	2.84	3.762 (3)	171
O1—H1⋯O2^iv^	0.90 (3)	1.88 (3)	2.7663 (17)	169 (3)
N2—H2⋯O1	0.87 (4)	2.23 (3)	3.0184 (19)	150 (3)

Symmetry codes: (i) 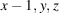; (ii) 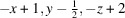; (iii) 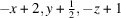; (iv) 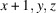.

**Table 2:** Experimental details

Crystal data	
Chemical formula	C_19_H_24_N_2_O_2_
*M*_r_	312.40
Crystal system, space group	Monoclinic, *P*2_1_
Temperature (K)	295
*a*, *b*, *c* (Å)	7.3745 (2), 10.6486 (2), 11.1872 (2)
β (°)	98.727 (1)
*V* (Å^3^)	868.34 (3)
*Z*	2
Radiation type	Cu *K*α
μ (mm^−1^)	0.62
Crystal size (mm)	0.3 × 0.25 × 0.05

Data collection	
Diffractometer	Bruker D8 VENTURE dual wavelength Mo/Cu
Absorption correction	Multi-scan (*SADABS*; Krause *et al.*, 2015 ▸)
*T*_min_, *T*_max_	0.63, 0.97
No. of measured, independent and observed [*I* > 2σ(*I*)] reflections	26843, 3310, 3234
*R*_int_	0.028
(sin θ/λ)_max_ (Å^−1^)	0.617

Refinement	
*R*[*F*^2^ > 2σ(*F*^2^)], *wR*(*F*^2^), *S*	0.033, 0.090, 1.05
No. of reflections	3310
No. of parameters	218
No. of restraints	1
H-atom treatment	H atoms treated by a mixture of independent and constrained refinement
Δρ_max_, Δρ_min_ (e Å^−3^)	0.14, −0.14
Absolute structure	Flack *x* determined using quotients [(*I*^+^)−(*I*^−^)]/[(*I*^+^)+(*I*^−^)] (Parsons *et al.*, 2013 ▸)
Absolute structure parameter	0.08 (5)

Computer programs: *APEX5* and *SAINT* (Bruker, 2016 ▸), *SHELXT2018/2* (Sheldrick, 2015*a* ▸), *SHELXL2018/3* (Sheldrick, 2015*b* ▸) and *OLEX2* (Dolomanov *et al.*, 2009 ▸).

## full crystallographic data

### 2-{[(1S,2S)-1-Hydroxy-1-phenylpropan-2-yl](methyl)amino}-N-(3-methylphenyl)acetamide Crystal data

**Table:**

C_19_H_24_N_2_O_2_	*F*(000) = 336
*M**_r_* = 312.40	*D*_x_ = 1.195 Mg m^−^^3^
Monoclinic, *P*2_1_	Cu *K*α radiation, λ = 1.54178 Å
*a* = 7.3745 (2) Å	Cell parameters from 9843 reflections
*b* = 10.6486 (2) Å	θ = 4.2–72.1°
*c* = 11.1872 (2) Å	µ = 0.62 mm^−^^1^
β = 98.727 (1)°	*T* = 295 K
*V* = 868.34 (3) Å^3^	Prism, colourless
*Z* = 2	0.3 × 0.25 × 0.05 mm

### 2-{[(1S,2S)-1-Hydroxy-1-phenylpropan-2-yl](methyl)amino}-N-(3-methylphenyl)acetamide Data collection

**Table:**

Bruker D8 VENTURE dual wavelength Mo/Cu diffractometer	3310 independent reflections
Radiation source: microfocus sealed X-ray tube, INCOATEC IµS	3234 reflections with *I* > 2σ(*I*)
Detector resolution: 7.3910 pixels mm^-1^	*R*_int_ = 0.028
φ and ω scans	θ_max_ = 72.1°, θ_min_ = 7.4°
Absorption correction: multi-scan (SADABS; Krause *et al.*, 2015)	*h* = −9→9
*T*_min_ = 0.63, *T*_max_ = 0.97	*k* = −13→13
26843 measured reflections	*l* = −13→13

### 2-{[(1S,2S)-1-Hydroxy-1-phenylpropan-2-yl](methyl)amino}-N-(3-methylphenyl)acetamide Refinement

**Table:**

Refinement on *F*^2^	Hydrogen site location: mixed
Least-squares matrix: full	H atoms treated by a mixture of independent and constrained refinement
*R*[*F*^2^ > 2σ(*F*^2^)] = 0.033	*w* = 1/[σ^2^(*F*_o_^2^) + (0.062*P*)^2^ + 0.0428*P*] where *P* = (*F*_o_^2^ + 2*F*_c_^2^)/3
*wR*(*F*^2^) = 0.090	(Δ/σ)_max_ < 0.001
*S* = 1.05	Δρ_max_ = 0.14 e Å^−^^3^
3310 reflections	Δρ_min_ = −0.14 e Å^−^^3^
218 parameters	Extinction correction: SHELXL-2016/6 (Sheldrick 2015a), Fc^*^=kFc[1+0.001xFc^2^λ^3^/sin(2θ)]^-1/4^
1 restraint	Extinction coefficient: 0.017 (3)
Primary atom site location: dual	Absolute structure: Flack *x* determined using ???? quotients [(*I*^+^)-(*I*^-^)]/[(*I*^+^)+(*I*^-^)] (Parsons *et al.*, 2013)
Secondary atom site location: difference Fourier map	Absolute structure parameter: 0.08 (5)

### 2-{[(1S,2S)-1-Hydroxy-1-phenylpropan-2-yl](methyl)amino}-N-(3-methylphenyl)acetamide Special details

**Table:**

Geometry. All esds (except the esd in the dihedral angle between two l.s. planes) are estimated using the full covariance matrix. The cell esds are taken into account individually in the estimation of esds in distances, angles and torsion angles; correlations between esds in cell parameters are only used when they are defined by crystal symmetry. An approximate (isotropic) treatment of cell esds is used for estimating esds involving l.s. planes.
Refinement. Hydrogen atoms attached to both O and N were refined isotropically. All C—H hydrogen atoms were refined with a riding model and thermal parameters at 1.2*U*_eq_(C) [1.5*U*_eq_ for CH_3_ groups].

### 2-{[(1S,2S)-1-Hydroxy-1-phenylpropan-2-yl](methyl)amino}-N-(3-methylphenyl)acetamide Fractional atomic coordinates and isotropic or equivalent isotropic displacement parameters (Å^2^)

**Table:**

	*x*	*y*	*z*	*U*_iso_*/*U*_eq_	
O2	0.19659 (16)	0.49733 (14)	0.75216 (13)	0.0570 (3)	
O1	0.83687 (17)	0.56561 (15)	0.67162 (13)	0.0621 (4)	
N2	0.50958 (18)	0.49800 (15)	0.79114 (13)	0.0444 (3)	
N1	0.5411 (2)	0.71980 (16)	0.68727 (15)	0.0541 (4)	
C12	0.3453 (2)	0.54641 (18)	0.74646 (15)	0.0444 (4)	
C11	0.3562 (3)	0.6750 (2)	0.6896 (2)	0.0628 (5)	
H11A	0.291810	0.672125	0.607361	0.075*	
H11B	0.292958	0.734947	0.733978	0.075*	
C8	0.6074 (2)	0.70207 (18)	0.57142 (16)	0.0490 (4)	
H8	0.544529	0.627981	0.533165	0.059*	
C7	0.8137 (2)	0.67175 (18)	0.59309 (17)	0.0493 (4)	
H7	0.880277	0.743225	0.633818	0.059*	
C10	0.5830 (4)	0.8383 (3)	0.7480 (2)	0.0760 (7)	
H10A	0.710747	0.857139	0.750148	0.114*	
H10B	0.555850	0.833006	0.829119	0.114*	
H10C	0.510220	0.903462	0.705160	0.114*	
C9	0.5630 (3)	0.8113 (3)	0.4835 (2)	0.0727 (7)	
H9A	0.590232	0.787704	0.405341	0.109*	
H9B	0.635548	0.882956	0.512560	0.109*	
H9C	0.435158	0.831937	0.477323	0.109*	
C13	0.5518 (2)	0.38461 (16)	0.85436 (14)	0.0422 (4)	
C14	0.7304 (2)	0.37236 (18)	0.91462 (16)	0.0473 (4)	
H14	0.811827	0.438983	0.914194	0.057*	
C15	0.7893 (3)	0.2626 (2)	0.97539 (16)	0.0539 (5)	
C16	0.6655 (3)	0.1640 (2)	0.97430 (18)	0.0614 (5)	
H16	0.702696	0.089149	1.013278	0.074*	
C17	0.4875 (3)	0.1765 (2)	0.9158 (2)	0.0639 (5)	
H17	0.405820	0.110039	0.916814	0.077*	
C18	0.4286 (3)	0.28598 (19)	0.85571 (17)	0.0539 (4)	
H18	0.308415	0.293557	0.816781	0.065*	
C19	0.9830 (3)	0.2510 (3)	1.0389 (2)	0.0777 (7)	
H19A	0.984099	0.205491	1.113103	0.117*	
H19B	1.033580	0.333206	1.056255	0.117*	
H19C	1.055267	0.206803	0.987990	0.117*	
C6	0.8869 (2)	0.64569 (17)	0.47647 (16)	0.0475 (4)	
C5	0.8472 (3)	0.53396 (19)	0.4147 (2)	0.0636 (5)	
H5	0.774224	0.474589	0.445596	0.076*	
C4	0.9139 (4)	0.5088 (2)	0.3080 (3)	0.0770 (7)	
H4	0.883943	0.433812	0.267074	0.092*	
C3	1.0244 (3)	0.5950 (3)	0.2629 (2)	0.0734 (6)	
H3	1.073542	0.577169	0.193008	0.088*	
C2	1.0624 (3)	0.7081 (3)	0.3215 (2)	0.0714 (6)	
H2A	1.135170	0.767248	0.290147	0.086*	
C1	0.9926 (3)	0.7334 (2)	0.42666 (19)	0.0596 (5)	
H1A	1.016741	0.810609	0.464675	0.072*	
H1	0.957 (4)	0.551 (3)	0.691 (3)	0.089*	
H2	0.604 (4)	0.541 (3)	0.775 (3)	0.089*	

### 2-{[(1S,2S)-1-Hydroxy-1-phenylpropan-2-yl](methyl)amino}-N-(3-methylphenyl)acetamide Atomic displacement parameters (Å^2^)

**Table:**

	*U*^11^	*U*^22^	*U*^33^	*U*^12^	*U*^13^	*U*^23^
O2	0.0337 (6)	0.0634 (8)	0.0729 (9)	0.0048 (6)	0.0043 (5)	0.0058 (6)
O1	0.0357 (6)	0.0814 (10)	0.0704 (8)	0.0125 (6)	0.0123 (5)	0.0361 (8)
N2	0.0342 (7)	0.0513 (7)	0.0483 (8)	0.0043 (6)	0.0076 (5)	0.0052 (6)
N1	0.0414 (7)	0.0595 (9)	0.0629 (9)	0.0089 (7)	0.0122 (6)	0.0127 (7)
C12	0.0348 (7)	0.0554 (9)	0.0431 (8)	0.0081 (7)	0.0068 (5)	−0.0011 (7)
C11	0.0388 (9)	0.0720 (13)	0.0795 (13)	0.0164 (8)	0.0152 (8)	0.0251 (10)
C8	0.0373 (8)	0.0533 (9)	0.0552 (10)	0.0063 (7)	0.0031 (6)	0.0043 (8)
C7	0.0358 (8)	0.0572 (10)	0.0544 (10)	0.0006 (7)	0.0047 (6)	0.0135 (7)
C10	0.0647 (14)	0.0934 (18)	0.0680 (14)	0.0043 (12)	0.0045 (10)	−0.0130 (12)
C9	0.0660 (13)	0.0922 (17)	0.0605 (13)	0.0334 (12)	0.0115 (9)	0.0251 (11)
C13	0.0402 (8)	0.0496 (9)	0.0377 (8)	0.0061 (6)	0.0092 (6)	0.0001 (6)
C14	0.0420 (8)	0.0559 (10)	0.0438 (8)	0.0051 (7)	0.0061 (6)	−0.0011 (7)
C15	0.0543 (10)	0.0677 (11)	0.0399 (9)	0.0173 (8)	0.0077 (7)	0.0032 (8)
C16	0.0748 (13)	0.0602 (11)	0.0520 (10)	0.0154 (10)	0.0187 (9)	0.0154 (8)
C17	0.0663 (12)	0.0608 (12)	0.0675 (13)	−0.0036 (10)	0.0192 (9)	0.0128 (9)
C18	0.0452 (9)	0.0620 (11)	0.0550 (10)	−0.0012 (8)	0.0091 (7)	0.0065 (8)
C19	0.0674 (14)	0.0945 (18)	0.0653 (13)	0.0278 (13)	−0.0091 (10)	0.0052 (12)
C6	0.0332 (7)	0.0517 (9)	0.0577 (10)	0.0047 (7)	0.0070 (6)	0.0163 (8)
C5	0.0631 (11)	0.0468 (10)	0.0875 (15)	0.0022 (8)	0.0327 (10)	0.0129 (9)
C4	0.0893 (16)	0.0541 (11)	0.0959 (17)	0.0085 (12)	0.0412 (14)	0.0010 (11)
C3	0.0699 (13)	0.0806 (15)	0.0765 (14)	0.0155 (12)	0.0330 (11)	0.0178 (12)
C2	0.0588 (12)	0.0888 (16)	0.0687 (13)	−0.0124 (11)	0.0163 (9)	0.0280 (12)
C1	0.0539 (10)	0.0637 (11)	0.0605 (11)	−0.0135 (9)	0.0059 (8)	0.0151 (9)

### 2-{[(1S,2S)-1-Hydroxy-1-phenylpropan-2-yl](methyl)amino}-N-(3-methylphenyl)acetamide Geometric parameters (Å, º)

**Table:**

O2—C12	1.225 (2)	C13—C18	1.390 (3)
O1—C7	1.426 (2)	C14—H14	0.9300
O1—H1	0.90 (3)	C14—C15	1.389 (3)
N2—C12	1.341 (2)	C15—C16	1.390 (3)
N2—C13	1.410 (2)	C15—C19	1.501 (3)
N2—H2	0.87 (4)	C16—H16	0.9300
N1—C11	1.449 (2)	C16—C17	1.381 (3)
N1—C8	1.465 (2)	C17—H17	0.9300
N1—C10	1.444 (3)	C17—C18	1.383 (3)
C12—C11	1.517 (3)	C18—H18	0.9300
C11—H11A	0.9700	C19—H19A	0.9600
C11—H11B	0.9700	C19—H19B	0.9600
C8—H8	0.9800	C19—H19C	0.9600
C8—C7	1.538 (2)	C6—C5	1.385 (3)
C8—C9	1.526 (3)	C6—C1	1.386 (3)
C7—H7	0.9800	C5—H5	0.9300
C7—C6	1.512 (3)	C5—C4	1.384 (3)
C10—H10A	0.9600	C4—H4	0.9300
C10—H10B	0.9600	C4—C3	1.374 (4)
C10—H10C	0.9600	C3—H3	0.9300
C9—H9A	0.9600	C3—C2	1.379 (4)
C9—H9B	0.9600	C2—H2A	0.9300
C9—H9C	0.9600	C2—C1	1.381 (3)
C13—C14	1.392 (2)	C1—H1A	0.9300
			
C7—O1—H1	108 (2)	C18—C13—N2	123.90 (15)
C12—N2—C13	129.35 (15)	C18—C13—C14	119.72 (16)
C12—N2—H2	115 (2)	C13—C14—H14	119.4
C13—N2—H2	116 (2)	C15—C14—C13	121.27 (18)
C11—N1—C8	114.52 (17)	C15—C14—H14	119.4
C10—N1—C11	114.43 (18)	C14—C15—C16	118.34 (17)
C10—N1—C8	116.92 (18)	C14—C15—C19	120.4 (2)
O2—C12—N2	125.56 (17)	C16—C15—C19	121.3 (2)
O2—C12—C11	120.75 (15)	C15—C16—H16	119.7
N2—C12—C11	113.64 (15)	C17—C16—C15	120.52 (18)
N1—C11—C12	114.48 (15)	C17—C16—H16	119.7
N1—C11—H11A	108.6	C16—C17—H17	119.4
N1—C11—H11B	108.6	C16—C17—C18	121.1 (2)
C12—C11—H11A	108.6	C18—C17—H17	119.4
C12—C11—H11B	108.6	C13—C18—H18	120.5
H11A—C11—H11B	107.6	C17—C18—C13	119.03 (17)
N1—C8—H8	106.9	C17—C18—H18	120.5
N1—C8—C7	110.02 (14)	C15—C19—H19A	109.5
N1—C8—C9	113.83 (16)	C15—C19—H19B	109.5
C7—C8—H8	106.9	C15—C19—H19C	109.5
C9—C8—H8	106.9	H19A—C19—H19B	109.5
C9—C8—C7	111.84 (16)	H19A—C19—H19C	109.5
O1—C7—C8	106.68 (14)	H19B—C19—H19C	109.5
O1—C7—H7	108.9	C5—C6—C7	120.80 (16)
O1—C7—C6	111.17 (15)	C5—C6—C1	117.90 (18)
C8—C7—H7	108.9	C1—C6—C7	121.29 (19)
C6—C7—C8	112.15 (14)	C6—C5—H5	119.3
C6—C7—H7	108.9	C4—C5—C6	121.38 (19)
N1—C10—H10A	109.5	C4—C5—H5	119.3
N1—C10—H10B	109.5	C5—C4—H4	120.2
N1—C10—H10C	109.5	C3—C4—C5	119.7 (2)
H10A—C10—H10B	109.5	C3—C4—H4	120.2
H10A—C10—H10C	109.5	C4—C3—H3	120.1
H10B—C10—H10C	109.5	C4—C3—C2	119.9 (2)
C8—C9—H9A	109.5	C2—C3—H3	120.1
C8—C9—H9B	109.5	C3—C2—H2A	120.0
C8—C9—H9C	109.5	C3—C2—C1	120.0 (2)
H9A—C9—H9B	109.5	C1—C2—H2A	120.0
H9A—C9—H9C	109.5	C6—C1—H1A	119.5
H9B—C9—H9C	109.5	C2—C1—C6	121.0 (2)
C14—C13—N2	116.35 (15)	C2—C1—H1A	119.5
			
O2—C12—C11—N1	−178.86 (18)	C10—N1—C8—C9	−48.9 (2)
O1—C7—C6—C5	45.4 (2)	C9—C8—C7—O1	−177.46 (17)
O1—C7—C6—C1	−135.88 (17)	C9—C8—C7—C6	−55.5 (2)
N2—C12—C11—N1	3.4 (3)	C13—N2—C12—O2	−0.8 (3)
N2—C13—C14—C15	−177.17 (15)	C13—N2—C12—C11	176.84 (17)
N2—C13—C18—C17	176.76 (17)	C13—C14—C15—C16	0.3 (3)
N1—C8—C7—O1	55.0 (2)	C13—C14—C15—C19	179.49 (18)
N1—C8—C7—C6	176.94 (16)	C14—C13—C18—C17	−1.0 (3)
C12—N2—C13—C14	−165.19 (17)	C14—C15—C16—C17	−1.1 (3)
C12—N2—C13—C18	17.0 (3)	C15—C16—C17—C18	0.8 (3)
C11—N1—C8—C7	−144.66 (16)	C16—C17—C18—C13	0.2 (3)
C11—N1—C8—C9	88.9 (2)	C18—C13—C14—C15	0.7 (3)
C8—N1—C11—C12	98.9 (2)	C19—C15—C16—C17	179.7 (2)
C8—C7—C6—C5	−73.9 (2)	C6—C5—C4—C3	1.1 (4)
C8—C7—C6—C1	104.8 (2)	C5—C6—C1—C2	−2.7 (3)
C7—C6—C5—C4	−179.8 (2)	C5—C4—C3—C2	−2.5 (4)
C7—C6—C1—C2	178.58 (18)	C4—C3—C2—C1	1.3 (4)
C10—N1—C11—C12	−122.2 (2)	C3—C2—C1—C6	1.3 (3)
C10—N1—C8—C7	77.5 (2)	C1—C6—C5—C4	1.5 (3)

### 2-{[(1S,2S)-1-Hydroxy-1-phenylpropan-2-yl](methyl)amino}-N-(3-methylphenyl)acetamide Hydrogen-bond geometry (Å, º)

**Table:**

*D*—H···*A*	*D*—H	H···*A*	*D*···*A*	*D*—H···*A*
C11—H11*A*···C1^i^	0.97	2.83	3.720 (3)	152
C16—H16···C12^ii^	0.93	2.80	3.378 (3)	121
C2—H2*A*···C14^iii^	0.93	2.86	3.687 (3)	149
C1—H1*A*···C5^iii^	0.93	2.84	3.762 (3)	171
O1—H1···O2^iv^	0.90 (3)	1.88 (3)	2.7663 (17)	169 (3)
N2—H2···O1	0.87 (4)	2.23 (3)	3.0184 (19)	150 (3)

Symmetry codes: (i) *x*−1, *y*, *z*; (ii) −*x*+1, *y*−1/2, −*z*+2; (iii) −*x*+2, *y*+1/2, −*z*+1; (iv) *x*+1, *y*, *z*.
